# Suppression of human colon tumor by EERAC through regulating Notch/DLL4/Hes pathway inhibiting angiogenesis *in vivo*

**DOI:** 10.7150/jca.61581

**Published:** 2021-08-08

**Authors:** Chenchen Yuan, Chenchen Wu, Rong Xue, Chun Jin, Chenguo Zheng

**Affiliations:** Department of Coloproctology, The Second Affiliated Hospital and Yuying Children's Hospital of Wenzhou Medical University, No 109 Xueyuan Western Road, Wenzhou, Zhejiang Province, 325000, P.R. China.

**Keywords:** CRC, Traditional Chinese medicine, Actinidia chinensis, Cancer, LDB2

## Abstract

**Background:** Ethanol extracted from radix of Actinidia chinensis (EERAC) has been proved to be effective to inhibit colorectal cancer (CRC). Notch signaling pathway and angiogenesis in tumors are closely related with the progression of CRC. However, if EERAC could influence CRC through Notch signaling pathway and angiogenesis remains unclear.

**Methods:** Flow cytometry, transwell, wound healing methods were used to measure cell apoptosis, invasion, migration, and proliferation. Protein and mRNA expression were detected using qRT-PCR and western blotting. Immunofluorescence staining was applied to detect the expression of target protein in the tissues.

**Results:** The invasion, migration, and proliferation of CRC cells were remarkably suppressed by ERRAC. Significant promotion of cell apoptosis and cell ration in S stage were observed after EERAC treatment. The Notch1/DLL4/Hes1 signaling pathway and angiogenesis were suppressed by EERAC. Overexpression of LIM domain-binding 2 (LDB2) remarkably weakened the influence of ERRAC on the viability of CRC cells.

**Conclusions:** EERAC might suppress CRC through targeting Notch/DLL4/Hes1 pathway and inhibiting angiogenesis in tumors. This study might provide novel thought for the prevention and therapy of CRC through targeting Notch/DLL4/Hes1.

## Introduction

With the change of living habits, the incidence rate and mortality of colorectal cancer (CRC) have been increasing recently, which has seriously affected people's health 1, 2. At present, the main treatment of CRC is surgery. However, due to no special symptoms in the early stage of CRC, most of CRC patients are found later, so postoperative recurrence or metastasis often leads to poor surgical results 3, 4. Gene therapy is the hope of conquering cancer 5. Studying the mechanism of CRC development from the molecular level may be able to screen out more effective therapeutic targets, so as to improve the cure rate and survival rate of patients.

Traditional Chinese medicine (TCM) has been proved to be able to exert its anti-cancer effect in many ways, targets and links, with less side effects, less drug resistance and low treatment cost 6, 7. Some TCMs have been commonly applied in the anti-tumor fields. Chinese herbal formulas Miao-Yi-Ai-Tang and Flavonoids was proved to be effective for lung cancer 8 and cervical carcinoma 9 treatment, respectively. Meanwhile, docetaxel has been believed to be effective for the treatment of ovarian and breast cancer 10-12.

Actinidia chinensis has been proved to act an anti-tumor role in some kinds of tumors such as gastric, liver, and breast cancer 13, 14. Several studies have confirmed the anti-tumor roles of ethanol extract from radix of Actinidia chinensis (EERAC) in different kinds of tumors. For example, EERAC could suppress hepatocellular carcinomas cells via LAMB3 15. The proliferation and metastasis of hepatocellular carcinoma could be inhibited by EERAC through inhibiting DLX2/TARBP2/JNK/AKT pathway and epithelial-mesenchymal transition 16, 17. Oligoadenylate synthetase L could regulate the lung cancer cell sensitivity through EERAC 18. In addition, we demonstrated that EERAC presented potential therapeutic function on CRC through Notch signaling pathway 19. However, the further targeting molecules and specific suppression mechanism have not been fully clarified.

Notch signaling pathway is a classic and highly conservative signal transduction system. Notch signaling pathway is mainly composed of receptor, ligand and DNA binding protein in nucleus 20. Notch has 4 receptors, which are notch 1-4. Notch ligands (Delta1, delta3, delta4, Jagged1 and jagged2) are single transmembrane glycoprotein widely distributed in extracellular, transmembrane and intracellular regions 21. The downstream gene of Notch signaling pathway include hairy and enhancer of split-1 (Hes 1). Notch protein itself does not have enzyme activity, and it acts a role in regulating cell proliferation, differentiation and apoptosis through the combination of receptor and ligand 22. However, its role in tumor is two-sided, and it can regulate both oncogene and tumor suppressor gene. Notch ligand Delta-like 4 (DLL4) has been proved to be closely related with tumorigenesis and tumor angiogenesis 23. If EERAC could affect CRC through Notch/DLL4 influencing tumor angiogenesis has not been reported.

In this study, we proved that EERAC remarkably inhibited CRC through suppressing Notch/DLL4/Hes1 pathway, and further inhibited tumor angiogenesis. This study might provide new insight about the prevention and therapy for CRC through targeting Notch/DLL4/Hes1 signaling pathway.

## Methods

### Isolation and preparation of EERAC

The EERAC used in this study was isolated in our laboratory (Patent publication No.: CN1977869A). Libermannn Burchard reaction and foam test were used to identify the main active chemical components of EERAC, which were triterpene saponins. 0.1% DMSO was used to dissolve EERAC, and 50, 100, 200 µg/mL EERAC were made. The study was approved by the Ethic Committee of the Second Affiliated Hospital and Yuying Children's Hospital of Wenzhou Medical University. All experiment protocols were in accordance with the Helsinki declaration.

### Cell culture

SW480 (colorectal cancer cell line) was purchased from Chinese Academy of Science (Beijing, China). The cells were cultivated in DMEM medium (#12430104, Gibco, Langley, OK, USA) containing 5% fetal bovine serum (FBS, #12483020, Gibco, USA), and cultured on the condition 5% CO_2_ and 37 °C. Then, the cells were treated with different concentration of ERRAC, and used for experiments. Overexpression of LIM domain-binding 2 (LDB2) was established through transfecting pCMV2-FLAG-LDB2 vector (2 μg), which was purchased from GenePharma Co., Ltd (Shanghai, China).

### CCK-8 assay

1×10^4^ cells were seeded firstly. After treatment with 50, 100, 200 µg/mL EERAC or pCMV2-FLAG-LDB2 vector (2 μg) for 48 h, the CCK-8 kit (#C0037, Beyotime, Beijing, China) was used to measure cell proliferation. 10 μL CCK-8 regent was added to each well, after 2 h incubation, OD at 450 nm was investigated.

### Transwell assay

3×10^5^ cells were plated on the top chamber firstly. Then, 300 μL medium containing 10% FBS (#12483020, Gibco, USA) was added to the bottom chamber. After 24 h, cells on the bottom were fixed using 4paraformaldehyde (%) for 20 min and stained using 0.1% crystal violet (#32675, Sigma, USA) for 20 min. Then, the invasive Cells were analyzed using an inverted microscope (BX53, Olympus, Japan) through calculating 3 random fields.

### Wound healing assay

4×10^5^ cells were plated into 6-well plates. After 12 h, 1 mL pipette tip was applied to drawn line in the middle of 6-well plates. Ensure the same width of each line as much as possible. Remove the medium and replace with new medium. The cells were cultured in the incubator of 5% CO_2_ and 37 °C. Cells were recorded at 0 h and 48 h was after scratching by taking photos. Then, the relative migrated distance was analyzed.

### Western blot

Cells were lysed with lysis buffer (#P0013K, Beyotime, Beijing, China), and proteins were extracted. Protein contents were detected using a BCA kit (#A045-4-2, Nanjing Jiancheng Bioengineering Institute, Nanjing, China). Then, 30 µg of protein was loaded for 8% SDS-PAGE, and transferred to a PVDF membrane (#03010040001, Millipore, Bedford, MA, USA). The membrane was blocked using Tris-Buffered Saline, 0.1% Tween (TBST) containing 5% non-fat milk for 2 h at room temperature. After washing 3 times, proteins were incubated with primary antibodies at 4 °C overnight followed by secondary antibodies for 2 h. Proteins were measured with an enhanced chemiluminescence detection kit (#SLF1022, Thermo, Waltham, MA, USA), and protein band analysis was conducted using ImageJ software. The antibodies details are listed below: Anti-notch 1 antibody (ab221603, Abcam Cambridge, UK), Anti-DLL4 antibody (ab176876, Abcam, UK), Anti-Hes1 antibody (ab108937, Abcam, UK), Anti-GAPDH antibody (ab181602, Abcam, UK), Goat polyclonal secondary antibody to rabbit IgG (ab150077, Abcam, UK).

### qRT-PCR

Total RNA was extracted by Trizol reagent (#RR024A, Takara, Beijing, China) in one step. The purity of RNA was determined by micro ultraviolet spectrophotometer, and OD 260 nm/280 nm was 1.91. cDNA was detected using real time PCR with ChamQ^TM^ SYBR^®^ qPCR Master Mix (Vazyme). The information of primers was listed in Table 1. 2^-ΔΔCT^ method was used in this study to analyze the gene expression.

### Flow cytometry

5×10^5^ cells were seeded into 6-well plates, and culture in the incubator. After different with with 50, 100, 200 µg/mL EERAC or pCMV2-FLAG-LDB2 vector (2μg) for 48 h, cells were digested. After centrifugation, the supernatant was removed, and the pellet was re-suspended with 500 µL PBS buffer containing propidium iodide (10 Μl, # C1062S, Beyotime) and Annexin V-FITC (10 μL, # C1062S, Beyotime). Then, the cells were incubated in the dark for 20 min, apoptosis was measured with flow cytometry.

### Immunohistochemical staining

After sacrifice of mice, the tumor tissues were fixed using 4% formalin. Then, the tissues were embedded with OCT, and cut into 6 μm sections using a freezing microtome. After antigen repair (5 min), PBS washing (twice, 3 min/time), blocking (5% goat serum), and PBS washing (twice, 3 min/time), tissues were cultured with primary antibody overnight. Then, tissues were incubated with secondary antibody for 3 h, and then cultured with DAPI for 15 min. Finally, slides were analyzed using Olympus BX41 microscope (Tokyo, Japan). The antibodies details are listed below: Anti-notch 1 antibody (ab221603, Abcam, UK), Anti-DLL4 antibody (ab176876, Abcam, UK), Anti-Hes1 antibody (ab108937, Abcam, UK).

### Statistical analysis

Results were presented as mean ±SD, and analyzed uisng SPSS software (22.0, IBM, USA). An unpaired 2-tailed Student's t-test was applied to compare the data of two groups. One-way ANOVA was used to analyze data in at least 3 groups. p <0.05 was considered to be statistical difference. Experiments were conducted at least 3 times.

## Results

### The invasion, migration, and proliferation of CRC cells were remarkably suppressed by ERRAC

**Figure 1:**
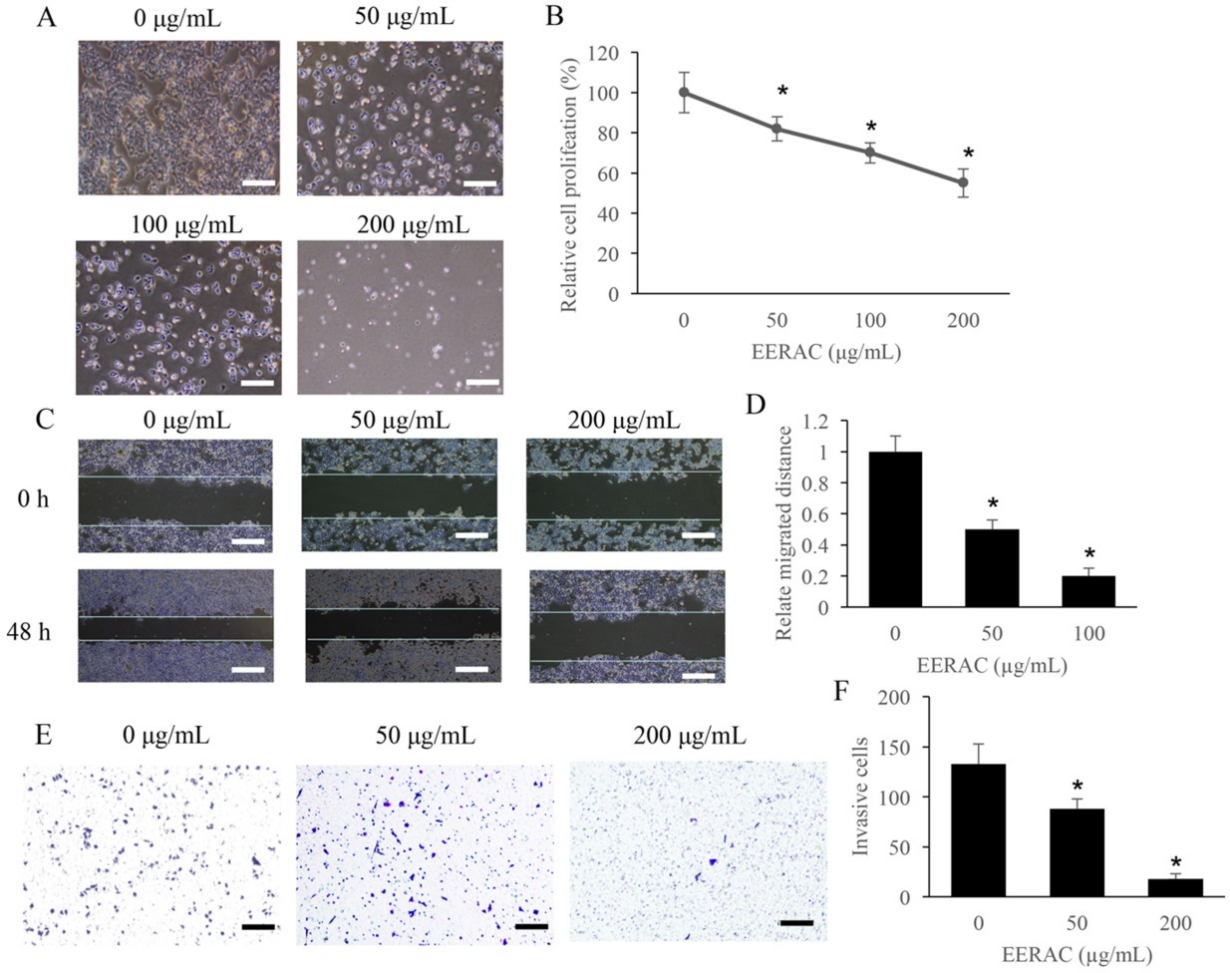
** The proliferation, migration, and invasion of CRC cells were remarkably suppressed by ERRAC. (A)** Cell morphology investigation after EERAC treatment (Scale bar: 300 µm). **(B)** ERRAC significantly inhibited the cell proliferation of SW480 cells. **(C)** Investigation of cell migration with wound healing assay (Scale bar: 500 µm). **(D)** ERRAC significantly inhibited the cell migration of SW480 cells. **(E)** Measurement of cell invasion with transwell assay (Scale bar: 400 µm). **(F)** ERRAC remarkably suppressed the cell invasion of SW480 cells. *P <0.05 compared with group 0 µg/mL EERAC. These experiments were performed 3 times independently.

The effect of ERRAC on the cell proliferation of SW480 cells was firstly investigated. Remarkable inhibition of cell proliferation after ERRAC treatment was observed, and the suppression effect was dose-dependent manner (Figure 1A-B). In addition, the cell migration of SW480 cells was also markedly suppressed by ERRAC (Figure 1C-D). In addition, both 50 µg/mL and 200 µg/mL ERRAC could markedly inhibit the cell invasion of CRC cells (Figure 1E-F).

### Significant promotion of cell apoptosis and cell ration in S stage were observed after EERAC treatment

**Figure 2:**
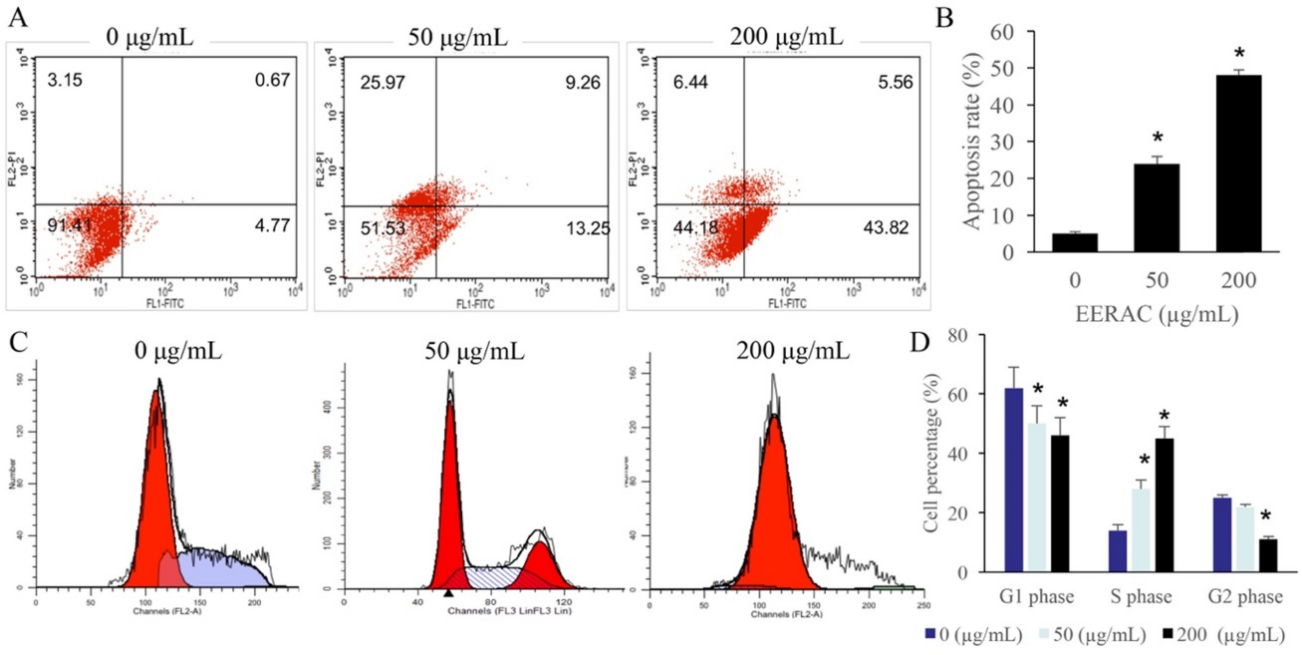
** Significant promotion of cell apoptosis and cell ration in S stage were observed after EERAC treatment. (A)** Cell apoptosis was detected with flow cytometry. **(B)** ERRAC significantly promoted the cell apoptosis of SW480 cells. **(C)** Cell cycle was detected with flow cytometry. **(D)** ERRAC remarkably increased the cell percentage in the S phase. *P <0.05 compared with group 0 µg/mL EERAC. These experiments were performed 3 times independently.

The cell apoptosis and cell cycle were observed after treatment with 0, 50, and 200 µg/mL ERRAC. Remarkable increase of SW480 cell apoptosis was found after treatment with either 50 or 200 µg/mL ERRAC compared with 0 µg/mL ERRAC (Figure 2A-B). Cell cycle data indicated that 50 and 200 µg/mL ERRAC remarkably increased the cell percentage in the S phase, but the cell ratio in the G2 and G1 stages was markedly suppressed after treatment with 200 µg/mL ERRAC (Figure 2C-D).

### The Notch1/DLL4/Hes signaling pathway was suppressed by EERAC

**Figure 3:**
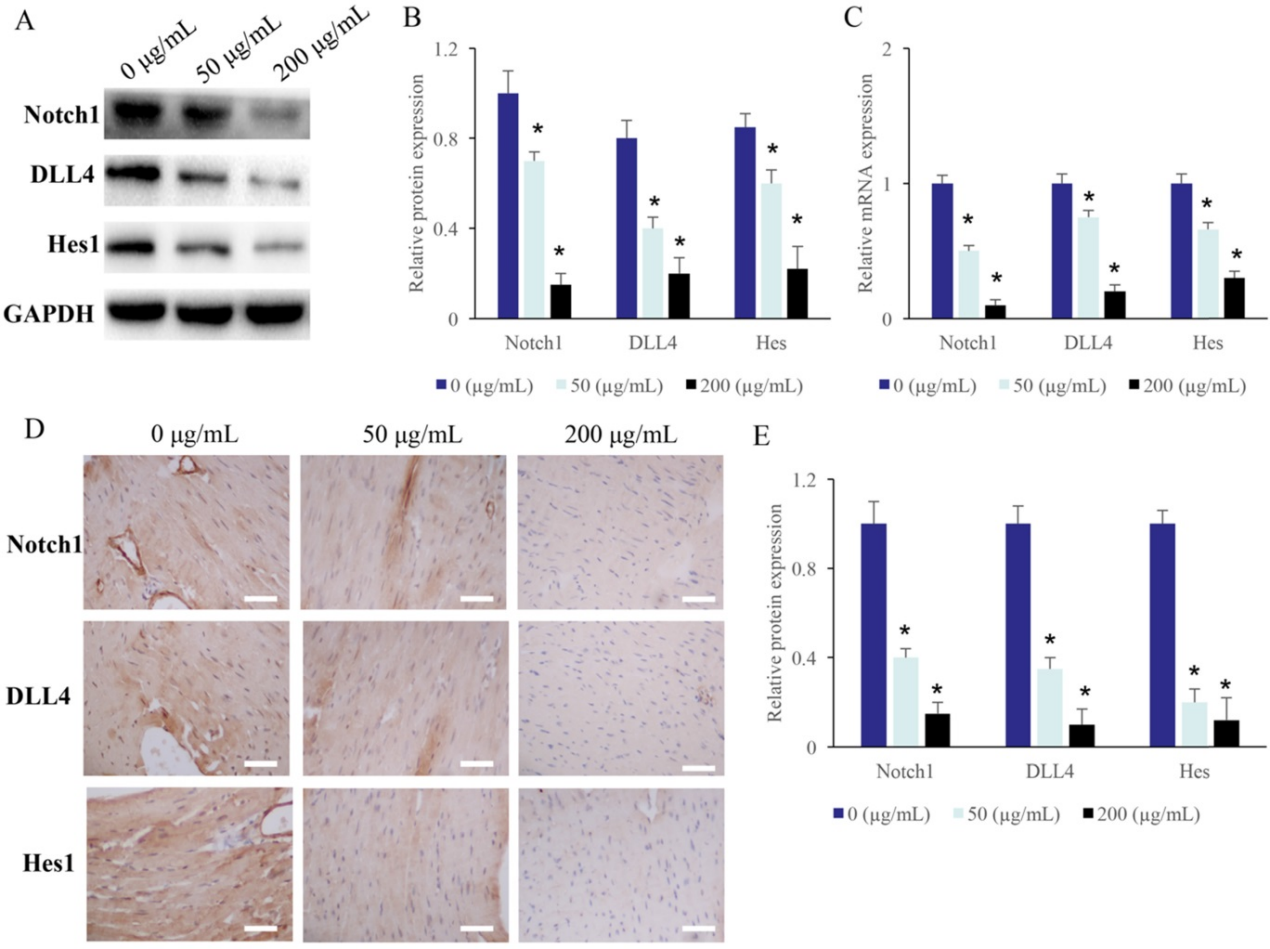
** The Notch1/DLL4/Hes signaling pathway was suppressed by EERAC. (A)** The protein expression of Notch1/DLL4/Hes in the CRC cells was measured with western blotting. **(B)** ERRAC remarkably inhibited the protein levels of Notch1, DLL4, and Hes in the CRC cells. **(C)** ERRAC remarkably inhibited the mRNA levels of Notch1, DLL4, and Hes in the CRC cells. **(D)** The protein expression of Notch1/DLL4/Hes in the tumor tissues was measured with IHC (Scale bar: 400 µm). **(E)** ERRAC remarkably inhibited the proteins levels of Notch1, DLL4, and Hes in the tumor tissues; *P <0.05 compared with group 0 µg/mL EERAC. These experiments were performed 3 times independently.

The protein levels of Hes, DLL4, and Notch1 in the CRC cells were measured after treatment with different concentrations of EERAC. After treatment with 50 and 200 µg/mL ERRAC, the protein and mRNA levels of Hes, DLL4, and Notch1 in the CRC cells were markedly suppressed (Figure 3A-C).

### The angiogenesis in the tumor tissues were markedly inhibited by EERAC

**Figure 4:**
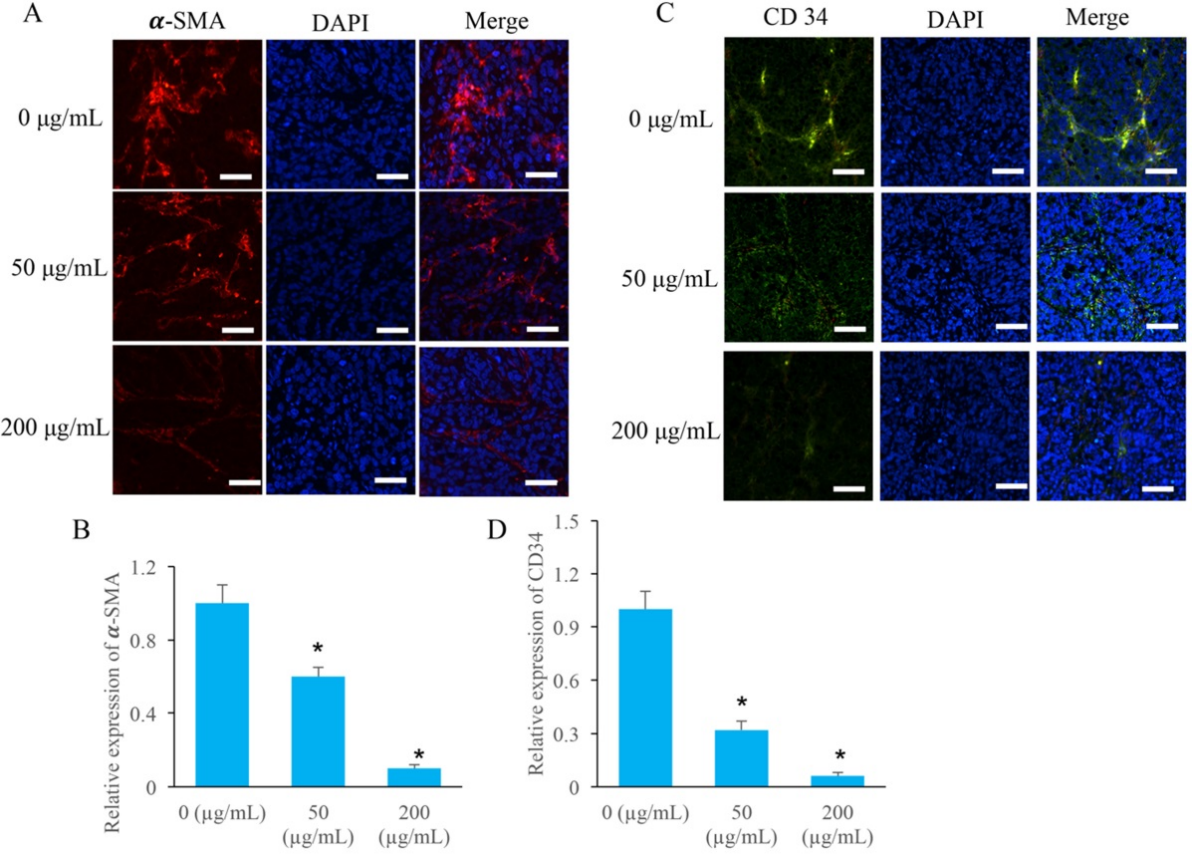
** The angiogenesis in the tumor tissues were markedly inhibited by EERAC. (A)** The level of α-SMA was measured using immunofluorescence staining (Scale bar: 100 µm). **(B)** ERRAC significantly inhibited the expression of α-SMA in the tumor tissues. **(C)** The level of CD-34 was measured using immunofluorescence staining (Scale bar: 100 µm). **(D)** ERRAC significantly inhibited the expression of CD34 in the tumor tissues. *P <0.05 compared with group 0 µg/mL EERAC. These experiments were performed 3 times independently.

Two special angiogenesis markers, α-SMA and CD34, in tumor tissues were detected after ERRAC treatment. After treatment with 50 and 200 µg/mL ERRAC, the level of α-SMA was remarkably suppressed compared with group 0 µg/mL ERRAC (Figure 4A-B). Similarly, 50 and 200 µg/mL ERRAC also significantly inhibited the expression of CD34 (Figure C-D).

### Overexpression of LDB2 remarkably weakened the influence of ERRAC on the cell viability of SW480 cells

**Figure 5:**
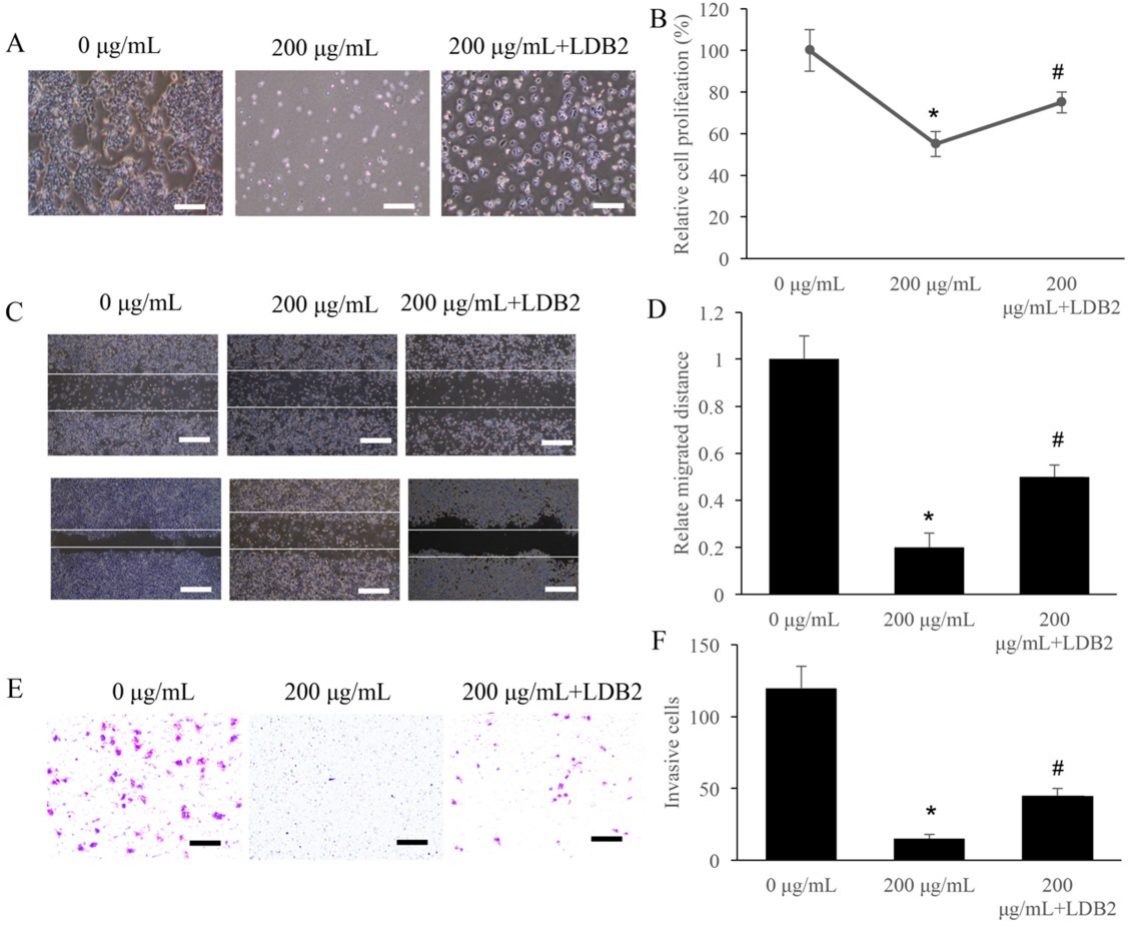
** Overexpression of LDB2 remarkably weakened the influence of ERRAC on the cell viability of SW480 cells. (A)** Cell morphology investigation after EERAC and overexpression of LDB2 treatment (Scale bar: 300 µm). **(B)** Overexpression of LDB2 significantly increased the cell proliferation of SW480 cells compared with group 200 µg/mL ERRAC. **(C)** Investigation of cell migration with wound healing assay (Scale bar: 500 µm). **(D)** Overexpression of LDB2 significantly promoted the cell migration of SW480 cells compared with group 200 µg/mL ERRAC. **(E)** Measurement of cell invasion with transwell assay (Scale bar: 100 µm). **(F)** Overexpression of LDB2 remarkably inhibited the cell invasion of SW480 cells compared with group 200 µg/mL ERRAC. *P <0.05 compared with group 0 µg/mL EERAC, # P<0.05 compared with group 200 µg/mL EERAC. These experiments were performed 3 times independently.

LDB2 is one of the activator of DLL4, and overexpression of LDB2 could increase the level of LDB2. We found that 200 µg/mL ERRAC remarkably suppressed the cell proliferation of SW480 cells, but overexpression of LDB2 significantly revised the influence of ERRAC, and increased cell proliferation (Figure 5A-B). Similarly, though 200 µg/mL ERRAC remarkably suppressed the cell migration (Figure 5C-D) and invasion (Figure 5E-F) of CRC cells, but simultaneous treatment with overexpression of LDB2 could remarkably promote the migration and invasion ability compared with group 200 µg/mL ERRAC only (Figure 5C-F).

### Overexpression of LDB2 remarkably revised the influence of ERRAC on the cell apoptosis and cell cycle of SW480 cells

**Figure 6:**
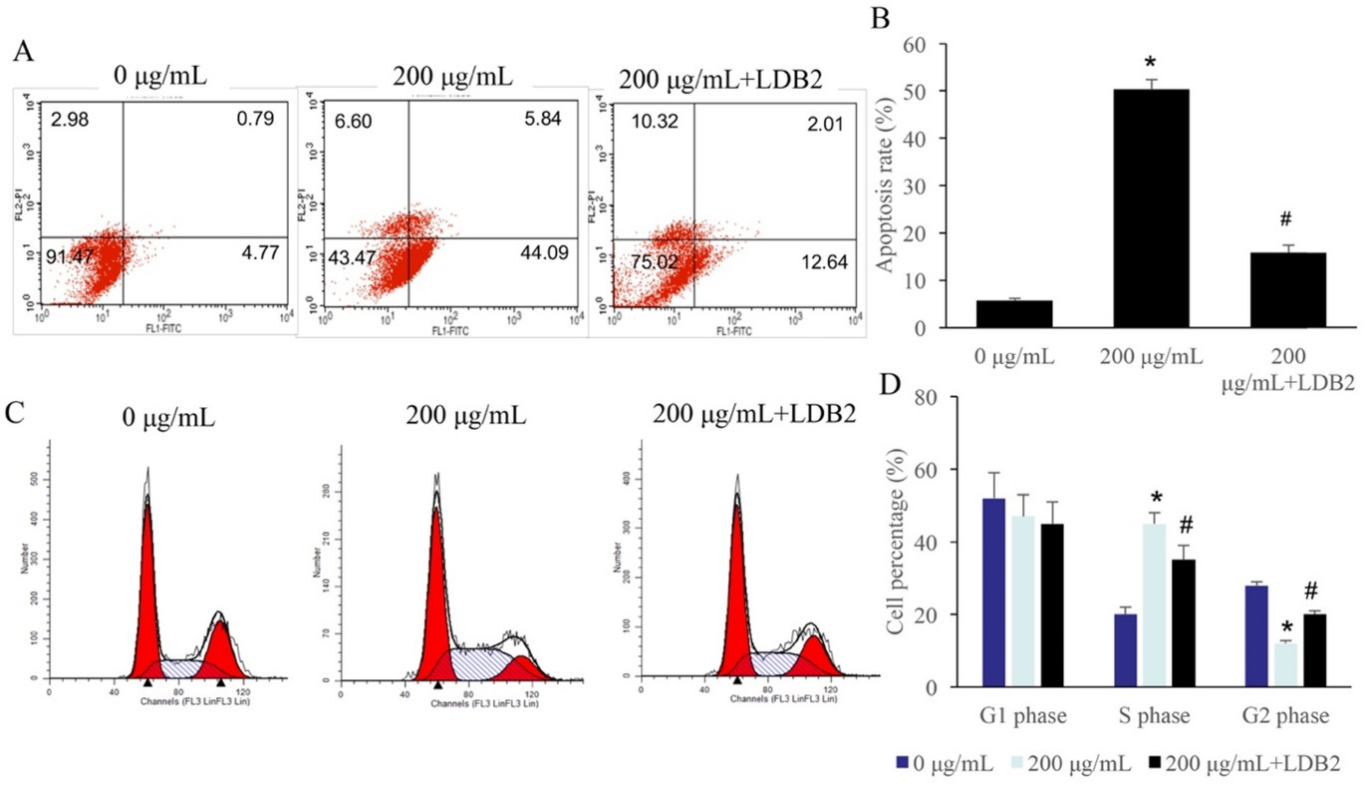
** Overexpression of LDB2 remarkably revised the influence of ERRAC on the cell apoptosis and cell cycle of SW480 cells. (A)** Cell apoptosis was detected with flow cytometry. **(B)** Overexpression of LDB2 significantly decreased the cell apoptosis of SW480 cells compared with group 200 µg/mL ERRAC. **(C)** Cell cycle was detected with flow cytometry. **(D)** Overexpression of LDB2 remarkably suppressed the cell percentage in the S phase compared with group 200 µg/mL ERRAC. *P <0.05 compared with group 0 µg/mL EERAC, # P<0.05 compared with group 200 µg/mL EERAC. These experiments were performed 3 times independently.

The apoptosis of SW480 cells was significantly promoted by 200 µg/mL ERRAC treatment (Figure 6A-B). However, simultaneous treatment with overexpression of LDB2 markedly decreased the level of cell apoptosis (Figure 6A-B). Meanwhile, significant increase of cell percentage in S phase and decrease in G2 phase were observed after 200 µg/mL ERRAC treatment. The overexpression of LDB2 reversed the influence of ERRAC on cell cycle, and inhibited cell percentage in S phase, increased cell ratio in G2 phase (Figure 6C-D).

## Discussion

Notch signaling pathway is involved in the regulation of cell growth, proliferation and inflammation. It has been widely recognized that Notch signaling is closely related with the initiation and progression of tumor 24. In this present study, ERRAC remarkably inhibited the cell viability of CRC cells. Meanwhile, the Notch/DLL4/Hes1 signaling pathway was markedly suppressed by ERRAC. Therefore, ERRAC might affect CRC cells through targeting Notch/DLL4/Hes1 signaling pathway.

The blood vessels around tumor are closely related to tumor microenvironment (TME). Tumor progression, resistance to treatment, invasion and metastasis are closely related to the interaction between tumor cells and TME 25. Tumor cells interact with TME through a variety of paracrine signals, among which Notch signal is considered to be one of the important signaling pathways 26. In colon cancer, the adjacent blood vessels activate the Notch signal of tumor cells and promote the metastasis of tumor cells.

Notch signaling could influence vascular budding by regulating the differentiation of endothelial cells into tip cells and stem cells through targeting endothelial growth factor receptor2 (VEGFR2) 27. DLL4, the ligand of Notch signal, plays an important role in this process. Overexpressed DLL4 in ECs could activate Notch in tumor cells, which further accelerate angiogenesis of tumor cells. Inhibition of DLL4expression can inhibit tumor growth 28. Overexpressed of DLL4 can increase the production of nonfunctional blood vessels, resulting in poor tumor metastasis 29. In addition, a positive correlation between DLL4 and VEGF was observed. After blocking VEGF, the expression of DLL4 in tumor vessels decreased rapidly, indicating that VEGF and Notch/DLL4 signaling pathway could influence each other 27. In this study, we found that the angiogenesis and DLL4 expression could be suppressed by ERRAC. Meanwhile, overexpression of LDB2 could increase the level of DLL4. Simultaneous treatment with ERRAC and LDB2 could reverse the influence of ERRAC on cell viability of CRC cells. Therefore, ERRAC might inhibit angiogenesis through down-regulating DLL4.

Hes1, a downstream target gene of Notch signaling system, belongs to bHLH gene family. Hes1 is involved in cell differentiation, which can maintain a variety of immature cells in undifferentiated state, and may acts as an oncogene in a variety of tumors 30, 31. Hes1 could promote tumor proliferation, migration and invasion by negatively regulating PTEN 32. Some studies have shown that Hes1 is highly expressed in pancreatic cancer cells 33. We found that ERRAC remarkably inhibited the expression of Hes1, which should be further analyzed.

In summary, we firstly demonstrated that ERRAC significantly suppressed the cell proliferation, invasion, migration, and promoted the cell apoptosis of CRC cells. Meanwhile, the Notch1/DLL4/Hes1 signaling pathway and angiogenesis were markedly by ERRAC. Activator of DLL4, LDB2, could reverse the influence of ERRAC on cell proliferation, invasion, migration, apoptosis and cell cycle of SW480 cells. Therefore, EERAC might suppress CRC through targeting Notch/DLL4/Hes1 pathway and inhibiting angiogenesis in tumors.

## Figures and Tables

**Table 1:** Clinic parameters of enrolled patients

Features	Number of patients
**Age**	
≤50	64
>50	98
**Sex**	
Male	102
Female	60
**Lymph node status**	
N0	78
N1	54
N2	30
**Tumor stage**	
I-II	118
III-IV	44

## Abbreviations

EERAC: Extracted from radix of Actinidia chinensis
CRC: Colorectal cancer
TCM: Traditional Chinese Medicines
DLL4: Notch ligand Delta-like 4
PBS: Phosphate buffer saline
FBS: Fetal bovine serum
CCK-8: Cell counting kit-8
TME: Tumor microenvironment
VEGFR2: Endothelial growth factor receptor2
